# Case Report: Dual-Energy Computed Tomography of Cardiac Changes in IgG4-Related Disease

**DOI:** 10.3389/fcvm.2022.792531

**Published:** 2022-03-04

**Authors:** Ying Wang, Hui Zhou, Ping Hu, Jie Zhao, Yitao Mao, Zhixiao Li, Xi Zhao

**Affiliations:** ^1^Department of Radiology, Xiangya Hospital, Central South University, Changsha, China; ^2^National Clinical Research Center for Geriatric Disorders, Xiangya Hospital, Central South University, Changsha, China; ^3^Siemens Healthineers China, Shanghai, China

**Keywords:** dual energy computed tomography (DECT), coronary computed tomography angiography (CCTA), myocardial ischemia (MI), myocardial fibrosis (MF), IgG4-related disease (IgG4-RD), coronary heart disease

## Abstract

**Background:**

Dual-energy computed tomography (DECT) is used in coronary plaque characterization, myocardial perfusion imaging, and pulmonary embolism diagnosis; however, there is no relevant research on DECT in IgG4-related diseases (IgG4-RD) involving the coronary artery. We are the first to report DECT findings of cardiac morphology and function in IgG4-RD.

**Patient Findings:**

Multimodality cardiovascular imaging from a 63-year-old male patient, who presented with IgG4-related pancreatitis, was analyzed. An iodine map and spectral curves were obtained from the DECT, which can help to distinguish between non-calcified plaques and IgG4 lesions of the coronary artery, noninvasive FFR_CT_ (fractional flow reserve derived from coronary computed tomography angiography) and ECV (extracellular volume fraction) demonstrated myocardial ischemia and myocardial fibrosis, respectively.

**Conclusion:**

The DECT can detect coronary artery tumor-like lesions caused by IgG4-RD and simultaneously assess the morphological, functional, and histological characteristics of the myocardium. This may help to guide individualized and timely treatment and avoid potentially life-threatening complications.

## Background

It is now generally believed that IgG4-related diseases (IgG4-RD) are systemic diseases that can affect multiple organs. Numerous studies have shown that coronary artery involvement is related to poor prognosis due to life-threatening complications such as myocardial infarction and aneurysmal rupture (1). Since it is difficult to obtain a coronary artery tissue biopsy, imaging plays an important role in the assessment of IgG4-related coronary disease. However, no imaging method can simultaneously distinguish IgG4 lesions from non-calcified plaques and analyze myocardial function. Considering the current applications of dual-energy computed tomography (DECT) in heart diagnostics, we present the first report of DECT findings in IgG4-related coronary disease.

## Case Presentation

A 63-year-old male presented with abdominal pain was admitted to our hospital. Laboratory examination showed that IgG4 (19.3 g/L) was elevated at nearly 8 times the upper limit of normal. Abdominal CT demonstrated a pancreatic mass and soft tissue lesions around the abdominal aorta and biliary tree. After completing a series of examinations and multidisciplinary discussions, the patient had a cumulative score of 38 points according to the inclusion criteria of the 2019 ACR/EULAR Classification Criteria for IgG4-RD (2) and was diagnosed with IgG4-RD. His past medical history included hypertension, type II diabetes, coronary artery disease with stable angina, and a splenectomy due to trauma. Coronary computed tomography angiography (CCTA) was conducted *via* DECT (SOMATOM Drive, Siemens Healthineers, Forchheim, Germany) and Syngo. *Via* workstation, “CT coronary artery,” “CT dual-energy,” and “CT cardiac function,” tools were used for measuring under the guidance of professional engineers. The circular regions of interest (ROI) were manually drawn to ensure that the ROIs were in the center of the lesions based on multiplanar three-dimensional reconstruction. The intra-observer and inter-observer intraclass correlation efficient (ICC) were 0.90 and 0.96, respectively. The measurements of deep learning-based fractional flow reserve derived from coronary computed tomography angiography (FFR_CT_) were performed by an independent core laboratory at Keya Medical (3).

**Figure 1:**
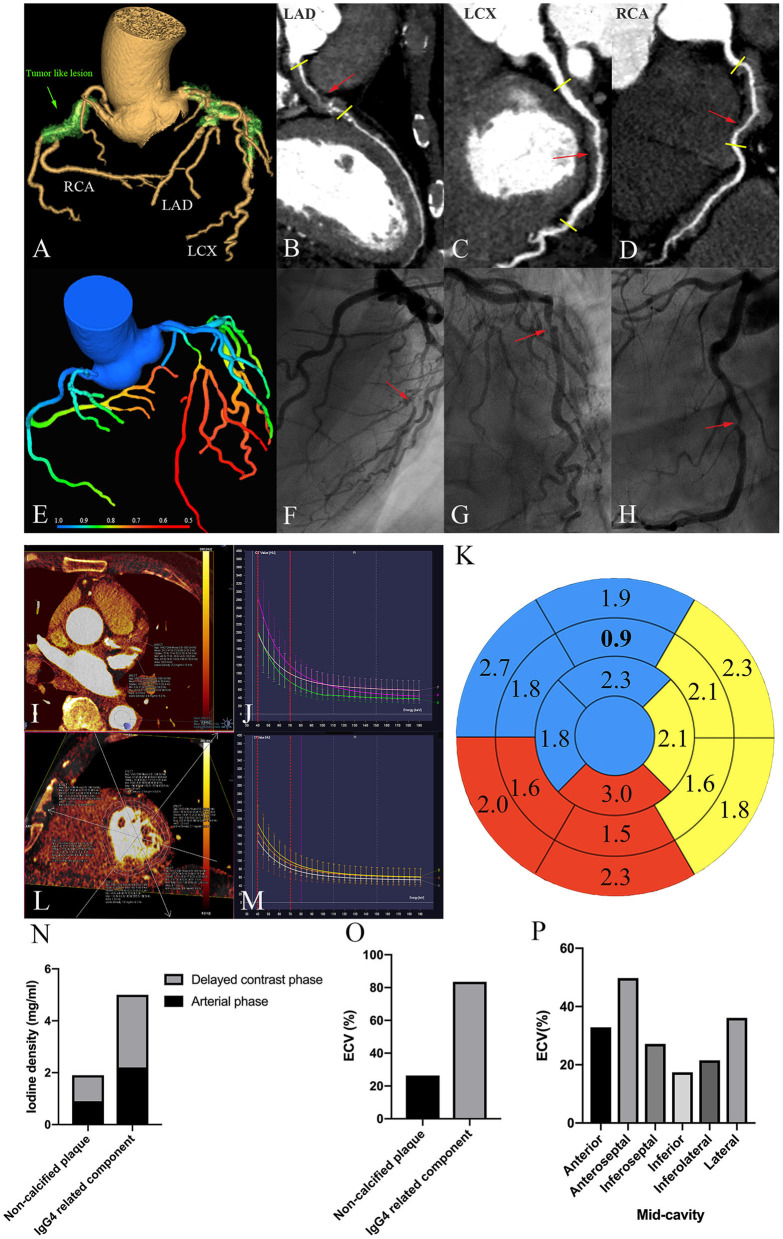
Multimodality imaging of IgG4-RD involving the coronary artery. **(A)** 3D volume-rendered-reconstructed CCTA demonstrates tumor-like lesions involving the RCA, LAD, and LCX (a green arrow). **(B–D)** Curved planar reformation of the CCTA. The red arrow indicates the most severe stenosis. The short yellow line shows the obvious arterial wall thickening within three vessels. **(E)** Myocardial ischemia shown by Fractional Flow Reserve (FFR) derived from CCTA. About 0.8 is the threshold for myocardial ischemia. **(F–H)** Invasive coronary angiogram. The red arrow shows the most severity stenosis corresponding to **(B–D)**. **(I)** Iodine density of non-calcified plaques and the IgG4 substance located in the proximal LAD in the arterial phase. **(N)** is the corresponding histogram. **(J)** The energy spectral curve in the arterial phase. Green: a non-calcified plaque; purple: An IgG4 substance; peach: septal myocardium. The reference points (40 and 70 Kev) are marked as a red-dotted line. **(K)** Bully's eye indicating iodine density in different myocardial segments. The blue, yellow, and red areas represent the myocardium dominated by LAD, LCX, and RCA, respectively. Bold number represents mid-anterior myocardium, which has the least iodine deposits. **(L)** Iodine density within mid-cavity myocardium. **(P)** is the corresponding histogram. **(M)** An energy spectral curve in the delayed contrast phase. White: a non-calcified plaques; yellow: an IgG4 substance; orange: septal myocardium. The reference points (40 Kev and 70 Kev) are marked as a red-dotted line. **(N)** Iodine density of the non-calcified plaques and the IgG4 substance in the arterial and delayed contrast phases. **(O)** ECV_CT_ of the non-calcified plaques and the IgG4 substance. **(P)** ECV_CT_ of the mid-cavity myocardium.

The CCTA showed multiple coronary arteries with moderate to severe stenosis lesions (Figures 1B–D); the left anterior-descending branch (LAD) was the most severely affected, which had 75–99% stenosis, the diagonal branch had 90% stenosis, the left circumflex branch (LCX) had 75–90% stenosis, and the right coronary artery (RCA) had 50–90% stenosis, which was confirmed *via* invasive coronary angiography (ICA) (Figures 1F–H). Interestingly, this patient had both non-calcified plaques and a lot of tumor-like lesions around the triple vessels (Figure 1A). The latter lesions might be caused by IgG4-RD, which were neglected during ICA. As we have known, IgG4-RD causes periarteritis that is predominantly affecting the adventitia, while the intima and media are less involved (1). However, non-calcified plaques are primarily located inside the lumen because it initially occurs in the intima of the coronary artery. The plaque inside the proximal LAD was easier to distinguish from the IgG4-related infiltration surrounding the coronary artery; therefore, we chose this area to measure the two lesions (Figure 1I). The mean CT attenuation value of the tumor-like lesion in proximal LAD was 38 HU in non-contrast images (Tube Voltage 100 keV), which was like the fibrous fatty plaque (45HU) located on the same slice of CT axial images. During the delayed contrast phase, the mean CT attenuation of the non-calcified plaque was 64 HU, while the tumor-like lesion was 100 HU. Combining with their enhanced features, we can further determine the non-calcified plaques and the IgG4-related infiltrates.

Firstly, we compared the iodine density of different lesions in LAD. In the arterial phase, tumor-like lesions took up more iodine than the non-calcified plaques, and the gap was widened with time (Figures 1I,N). The absolute and normalized iodine concentrations of the non-calcified plaques were 0.9 mg/ml and 5.2%; the IgG4-related components were 2.2 mg/ml and 12.8%, respectively. Normalized iodine concentration = plaque iodine concentration/aortic iodine concentration. Secondly, we compared the iodine density in different myocardial segments based on AHA segmentation (Figure 1L). The concentration of myocardial iodine, supplied by each coronary artery, can be seen on a Bull's eye plot (Figure 1K). The mid-anterior wall supplied by LAD had the least iodine deposits, indicating critical myocardial ischemia. The results of FFR_CT_ arrived at the same conclusion (Figure 1E). The most severely affected myocardium is supplied by LAD, followed by RCA and LCX. Referencing the FFR_CT_ threshold of myocardial ischemia (0.8), the FFR_CT_ value of the LAD was 0.58, which was much lower than LCX (0.75) and RCA (0.74). Subsequently, we calculated the myocardial ECV values. The iodine density-derived ECV was calculated as follows: ECV (%) = (1 − hematocrit) × (iodine density in myocardium)/(iodine density in the blood) × 100%. The IgG4-related component had higher ECV values compared to the non-calcified plaques (Figure 1O); the values of the mid-cavity myocardium are shown in Figure 1P. Meanwhile, the cardiac function analysis in CT showed that left ventricle ejection fraction (LVEF) was 72%, left ventricle end-diastolic volume index (LVEDVI) was 72 ml/m^2^, LV mass index was 82 g/m^2^, right ventricle ejection fraction (RVEF) was 44%, and the right ventricle end-diastolic volume index (RVEDVI) was 104 ml/m^2^, which suggested the normal biventricular volume and function. In addition, we made energy spectrum curves and calculated the slope values (Figures 1J,M). The CT values and the energy spectrum curves of the non-calcified plaques, the IgG4-related lesions, and the descending aorta were measured at the same level in the arterial and delayed stage. The CT values corresponding to 70 and 40 keV single energy points were selected as the reference points within the energy spectrum curve. The slope of the energy spectrum curve was calculated by the formula “(40–70 Kev)/(70–40),” 40, and 70 Kev, which were the CT values of the 40 and 70 keV energy points, respectively. The curves of the non-calcified plaques and the IgG4-related components were well-separated. In the arterial phase, the slopes of the non-calcified plaques and the IgG4 substance were 4 and 5.3, respectively, while, in the delayed contrast phase, the corresponding values were 1.3 and 2, respectively.

Through the comprehensive analysis of the patient's cardiac examination, he had indications of coronary stent implantation; however, considering the patient's older age, multiple complications, and high anesthesia risk, after multidisciplinary discussion and full communication with the patient and his family members, stent implantation was postponed, and the corticosteroid therapy (prednisone) was chosen as the first treatment. Other main treatments and corresponding medications included anticoagulation and antithrombosis (aspirin and clopidogrel), lowering lipid (atorvastatin), controlling blood sugar (metformin), controlling blood pressure, and protecting myocardium (irbesartan, metoprolol, and isosorbide mononitrate), etc. Meanwhile, the patient has been treated with particular caution and strict follow-up. The IgG4 level decreased from 19.3 to 3 g/L under systemic therapy within 7 months. The mass in the pancreas and soft tissue lesions around the biliary tree were reduced in size.

## Discussion

Given that IgG4-related coronary artery disease has fatal consequences, differential diagnosis has significant clinical importance. Because pathological findings of the coronary artery are hard to obtain, imaging plays a vital role in diagnosis due to its non-invasiveness and repetitiveness. Intravascular ultrasound, CCTA, and ^18^FDG-PET/CT are used to evaluate the lesions according to morphology. There are three types of coronary artery involvement: stenotic, aneurysmal, and diffuse wall thickening. As our case shows, the typical “mistletoe sign” in CCTA is a characteristic radiologic finding in IgG4-RD involving the coronary artery (4). However, the assessment of the disease's morphology and myocardial function cannot be performed concurrently using a single modality image. In our case, DECT solves this problem well. Iodine density mapping and spectral curve analysis can be used to analyze the components of IgG4-related coronary artery disease; FFR_CT_ and ECV can evaluate the degree of myocardial ischemia and myocardial fibrosis and may better guide clinical diagnosis and treatment.

The coronary plaque characteristics can be evaluated by measuring the CT attachment values; however, it is difficult to distinguish the tumor-like lesions from the non-calcified plaques on plain CT scans due to their similar CT attenuation values. The CCTA can detect periarteritis as a non-smooth soft tissue thickening around the arteries. One previous study shows that increasing iodine density after enhancement is related to perivascular inflammation (5). Coronary arteritis and periarteritis in IgG4-RD are primarily associated with adventitia, consisting of IgG4-positive plasma cell infiltration and fibrosis. The primary reason for the increase of iodine density within the tumor-like lesions after enhancement may be explained by coronary perivasculitis caused by IgG4-RD, which provides more information to help make a distinction. In recent years, intraluminal abnormalities and coronary artery aneurysms of IgG4-RD have been concerned (6, 7). We speculate that IgG4-RD induced coronary arteritis and vascular endothelial cell injury can lead to thrombosis and secondary lumen stenosis.

At the time of coronary angiography, FFR is the gold standard in evaluating hemodynamic significance of moderate coronary stenosis; however, FFR_CT_ offers a non-invasive and economical assessment. FFR_CT_ values less than or equal to 0.8 are considered as the threshold of myocardial ischemia (8). Myocardial fibrosis is an important indicator of myocardial damage and cardiac dysfunction. The ECV has been proved to correlate significantly with diffuse fibrosis within the myocardium (9). Emerging studies have found that, for detecting myocardial ECV changes, DECT is as useful as MRI (10). In our case, myocardial ischemia already existed in the rest situation, myocardial fibrosis has also occurred, and LAD contained the most severe lesions regardless of the non-calcified plaques or the IgG4-RD. It is interesting to note that the most obvious decrease of FFR_CT_ value was in the mid and distal LAD, but the most obvious decrease of iodine deposition was in the mid-anterior segment. We estimate that the collateral circulation formation in different segments is not alike, and the mid-segment may have the worst collateral circulation.

Since the previous study demonstrated that steroid therapy might lead to mild regression of the coronary stenosis on CCTA (11), it may also be possible to evaluate the efficacy of coronary periarteritis by comparing the changes of iodine deposition within the tumor-like lesions before and after steroid treatments, thus, providing clinical insight into the characterizations of plaques with an emphasis on the importance of early therapeutic intervention in IgG4-RD involving coronary arteries, such as corticosteroid therapy, surgery, immunomodulators, and immunosuppressants.

There are still some shortcomings in this study. Stress perfusion cannot be performed due to the lack of vasodilators and more cases, and prospective studies are needed to further validate our findings.

## CONCLUSION

The DECT provides a valuable basis for comprehensive assessments of IgG4-related infiltration, plaque composition, coronary stenosis, hemodynamic abnormality, cardiac function, and myocardial perfusion by one-step cardiac CT examination, especially for patients with MRI contraindications. Moreover, this wide range of information is beyond the reach of ICA. It is promising to be translated into clinical practice to improve the diagnosis and treatment of coronary artery disease of patients with IgG4-RD.

## Data Availability Statement

The original contributions presented in the study are included in the article/supplementary material, further inquiries can be directed to the corresponding author.

## Ethics Statement

The studies involving human participant was reviewed and approved by Ethics Committee of the Xiangya Hospital of Central South University. The patient/participant provided his written informed consent to participate in this study.

## Author Contributions

All authors listed have made a substantial, direct, and intellectual contribution to the work and approved it for publication.

## Funding

This work was supported by Natural Science Foundation of Hunan Province (2021JJ31131), China.

## Conflict of Interest

XZ is employed by Siemens Healthineers China. The remaining authors declare that the research was conducted in the absence of any commercial or financial relationships that could be construed as a potential conflict of interest.

## Publisher's Note

All claims expressed in this article are solely those of the authors and do not necessarily represent those of their affiliated organizations, or those of the publisher, the editors and the reviewers. Any product that may be evaluated in this article, or claim that may be made by its manufacturer, is not guaranteed or endorsed by the publisher.
